# Impact of Postarrest Vasoactive-Inotropic Score on Acute Kidney Injury in Cardiac Arrest Survivors: A Retrospective Cohort Study

**DOI:** 10.31083/j.rcm2501004

**Published:** 2024-01-04

**Authors:** Yu-Tzu Tien, Wen-Jone Chen, Chien-Hua Huang, Wei-Ting Chen, Hooi-Nee Ong, Tao-Ming Huang, Wei-Tien Chang, Min-Shan Tsai

**Affiliations:** ^1^Department of Emergency Medicine, National Taiwan University Medical College and Hospital, 100233 Taipei, Taiwan; ^2^Department of Internal Medicine (Cardiology Division), National Taiwan University Medical College and Hospital, 100233 Taipei, Taiwan; ^3^Department of Internal Medicine (Nephrology Division), National Taiwan University Medical College and Hospital, 100233 Taipei, Taiwan

**Keywords:** acute kidney injury, AKI staging, baseline kidney function, cardiac arrest, vasoactive inotropic score

## Abstract

**Background:**

Postarrest acute kidney injury (AKI) is a major health burden because it is associated with prolonged hospitalization, increased dialysis requirement, high mortality, and unfavorable neurological outcomes. Managing hemodynamic instability during the early postarrest period is critical; however, the role of quantified vasopressor dependence in AKI development in relation to illness severity remains unclear.

**Methods:**

A retrospective, observational cohort study that enrolled 411 non-traumatic adult cardiac arrest survivors without pre-arrest end-stage kidney disease between January 2017 and December 2019, grouped according to their baseline kidney function. The criteria for kidney injury were based on the Kidney Disease: Improving Global Outcomes definition and AKI staging system. The degree of vasopressor dependence within the first 24 h following return of spontaneous circulation (ROSC) was presented using the maximum vasoactive-inotropic score (${\mathrm{VIS}}_{\text{max}}$).

**Results:**

Of the 411 patients, 181 (44%) had early AKI after ROSC. Patients with AKI showed an increased risk of in-hospital mortality (adjusted OR [aOR] 5.40, 95% CI 3.36–8.69, *p* < 0.001) and unfavorable neurological outcome (aOR 5.70, 95% CI 3.45–9.43, *p* < 0.001) compared to patients without AKI. The risk of adverse outcomes increased with illness severity. Patients with vasopressor support had an increased risk of early AKI. A low ${\mathrm{VIS}}_{\text{max}}$ was associated with AKI stage 1–2 (aOR 2.51, 95% CI 1.20–5.24), whereas a high ${\mathrm{VIS}}_{\text{max}}$ was associated with an increased risk for AKI stage 3 (aOR 2.46, 95% CI 1.28–4.75).

**Conclusions:**

Early AKI is associated with an increased risk of in-hospital mortality and unfavorable neurologic recovery in cardiac arrest survivors. Postarrest ${\mathrm{VIS}}_{\text{max}}$ is an independent predictor of the development and severity of AKI following ROSC, regardless of baseline kidney function.

## 1. Introduction

Acute kidney injury (AKI) commonly arises as a complication in patients who have been successfully resuscitated from cardiac arrest (CA), with reported rates ranging from 12% to 81% [1, 2, 3, 4, 5]. Various factors contribute to this occurrence, including preexisting health conditions, reduced kidney perfusion during cardiopulmonary resuscitation (CPR), myocardial dysfunction, cardiovascular compromise following the return of spontaneous circulation (ROSC), and clinical interventions during the postarrest period [3, 5, 6]. Postarrest AKI poses a significant health burden due to its association with prolonged hospitalization, increased need for dialysis, elevated mortality rates, and poorer neurological outcomes [3, 5, 7]. Dutta *et al*. [3] reported that one-fifth of CA patients who developed AKI during hospitalization eventually required continuous kidney replacement therapy (KRT), with more than half necessitating dialysis even after discharge. Additional risk factors for postarrest AKI include male sex, advanced age, elevated baseline creatinine and urea levels, an initial nonshockable rhythm, and higher doses of vasoactive drugs and inotropes [2, 3, 4, 6].

Managing hemodynamic instability during the early postarrest period is critical. Patients who experienced out-of-hospital cardiac arrest (OHCA) and were on vasopressor support exhibited higher in-hospital mortality rates than those without such support [8]. Vasopressor usage is strongly associated with the development of postarrest AKI and an increased risk of long-term KRT [3, 4, 5, 9]. Vasoconstrictors can induce hemodynamic alterations and potentially worsen organ perfusion. Tujjar *et al*. [4] demonstrated a higher incidence of AKI among CA patients who received a larger cumulative epinephrine dose during resuscitation. The use of vasopressors following ROSC showed a strong correlation with AKI development and the continued need for dialysis post-discharge [3]. For pediatric patients with in-hospital cardiac arrest, the administration of multiple vasoactive agents within 24 h was identified as a risk factor for severe AKI [9]. However, studies investigating the association between vasopressor use and postarrest AKI have not yet quantified the extent of vasopressor administration. The vasoactive-inotropic score (VIS), which is a weighted sum of inotropes and vasoconstrictors administered in a specific period, reflects the overall pharmacological support of the cardiovascular system [10, 11]. The highest VIS value in 24 to 48 h has proven to be a valuable scoring system for predicting morbidity and mortality in patients with cardiac surgery and cardiac arrest [10, 11, 12]. Among surgical patients, the maximum VIS (${\mathrm{VIS}}_{\text{max}}$) during the initial 24 h stands as an independent predictor of postoperative AKI and a composite of unfavorable outcomes and long-term mortality [10, 13]. Nevertheless, the role of quantified vasopressor dependency in AKI development in relation to the severity of illness among adult cardiac arrest survivors remains unclear.

Hospitalized patients with an underlying impaired kidney function who subsequently developed AKI had poorer prognosis for morbidity and mortality compared to those with preserved or previously normal kidney function [14, 15, 16]. Furthermore, the severity of AKI was reported to be associated with in-hospital mortality regardless of baseline kidney function [14], with in-hospital mortality aligning more closely with AKI severity rather than preexisting chronic kidney disease [14, 17]. Thus, we aimed to assess the relationship between vasopressor dependency and the development of AKI following ROSC, as well as ascertain the significance of baseline kidney function in regard to the effect of vasopressor support on postarrest AKI.

## 2. Materials and Methods

### 2.1 Study Design and Setting

This retrospective, observational cohort study was conducted at National Taiwan University Hospital (NTUH), a 2500-bed tertiary medical center located in Taipei City (population density of approximately 10,000 people/km) with 110,000 annual emergency department (ED) visits [18]. The Institutional Review Board of the hospital approved the study (202203002RINB) and waived participant consent due to the nature of the study. Procedures were followed in accordance with the institutional ethical standards.

### 2.2 Data Collection

The primary dataset was sourced from the hospital medical records and included demographic information, past medical history, cardiac arrest events, postarrest management, laboratory examinations, and outcomes. This study adhered to the Strengthening the Reporting of Observational Studies in Epidemiology reporting guidelines [19].

Patients with cardiac arrest were categorized as either OHCA at a residential or public setting, including transfers from external hospitals or in-hospital cases after triage in the ED. An initial shockable rhythm was defined as the initial recorded rhythm being ventricular fibrillation or ventricular tachycardia. Repeated CPR was characterized as another arrest episode within 1 h after the initial ROSC. Cardiogenic arrest was recorded when the cause of arrest was attributed to ischemic heart disease, structural heart disease, heart failure, or arrhythmia without electrolyte imbalances. The determination of cardiac arrest causes was made by the responsible primary care physicians, who were blinded to the present study.

The lowest mean arterial pressure (MAP) during the initial 24 h following ROSC was categorized as ≥65 mmHg or <65 mmHg [20]. Patients with a pre-arrest Cerebral Performance Category (CPC) score of 1 or 2, no active bleeding or intracranial hemorrhage, and comatose consciousness after ROSC were eligible candidates for targeted temperature management (TTM). The TTM protocol at NTUH involved using cold saline and cooling devices with auto feedback to lower patients’ body temperatures to the targeted temperature of 33 °C within 4–6 h after ROSC. This targeted temperature was maintained for 24 h, followed by rewarming of patients at the rate of 0.25 °C per hour until 36 °C was achieved [21]. Temperature management to avoid fever was continued for another 24 h after rewarming. Instances of intra-aortic balloon pump and extracorporeal membrane oxygenation (ECMO) implantation were recorded if they occurred during the initial resuscitation. Emergent coronary angiogram and contrast-enhanced computed tomography scan were performed when indicated in patients within 24 h of ROSC [22]. Baseline laboratory test results at ROSC were documented. The levels of lactic acid (LA) in cardiac arrest survivors were categorized as <5 mmol/L, 5–10 mmol/L, and >10 mmol/L [8].

### 2.3 Predictor Variable

The highest amount of vasopressor use during the first 24 h of ROSC was denoted by ${\mathrm{VIS}}_{\text{max}}$, which reflects the degree of hypotension and severity of hemodynamic compromise during the early postarrest period. This was calculated as follows: dopamine dose (µg/kg/min) + dobutamine dose (µg/kg/min) + 100 × epinephrine dose (µg/kg/min) + 10 × milrinone dose (µg/kg/min) + 10,000 × vasopressin dose (unit/kg/min) + 100 × norepinephrine dose (µg/kg/min) using the maximum dosing rates of inotropic medications. The ${\mathrm{VIS}}_{\text{max}}$ was categorized into 3 groups: no ${\mathrm{VIS}}_{\text{max}}$, low ${\mathrm{VIS}}_{\text{max}}$ (≤30), and high ${\mathrm{VIS}}_{\text{max}}$ (>30) [23].

### 2.4 Outcome Measures

The primary outcome was the development of AKI during the early postarrest period. The criteria for diagnosing kidney injury were based on the Kidney Disease: Improving Global Outcomes (KDIGO) definition and AKI staging system characterized as an increase in serum creatinine by ≥0.3 mg/dL within 48 h [24]. AKI stage 3 was considered as severe AKI. Baseline creatinine levels were documented using records primarily from the previous 12 months or the admission creatinine, depending on the availability. Urine output volume was not used in this study. Secondary outcomes included the need for any modality of KRT during admission, in-hospital mortality, and poor neurological outcomes at hospital discharge. Poor neurological outcomes were defined as a CPC score of 3 (severe disability) to 5 (brain death).

### 2.5 Statistical Analysis

Categorical variables are presented using frequencies (percentages), while continuous variables are presented as medians (interquartile ranges). Comparisons were conducted using Fisher’s exact or Pearson’s chi-square test for categorical variables and the Mann-Whitney U test for continuous variables. Statistical significance was set at a* p*-value less than 0.05. Multiple imputation was used for missing data. Multiple logistic regression was performed to assess the associations between the predictor variable and outcomes, adjusted for variables with statistical significance and clinical relevance. Odds ratios (ORs) with 95% confidence intervals (CIs) were reported as an estimate of effect size and variability. Survival curves between groups were illustrated and compared using the log-rank test. All statistical analyses were performed using SPSS for Windows, version 16.0 (SPSS Inc., Chicago, IL, USA).

## 3. Results

### 3.1 Study Population

**Fig. 1.**
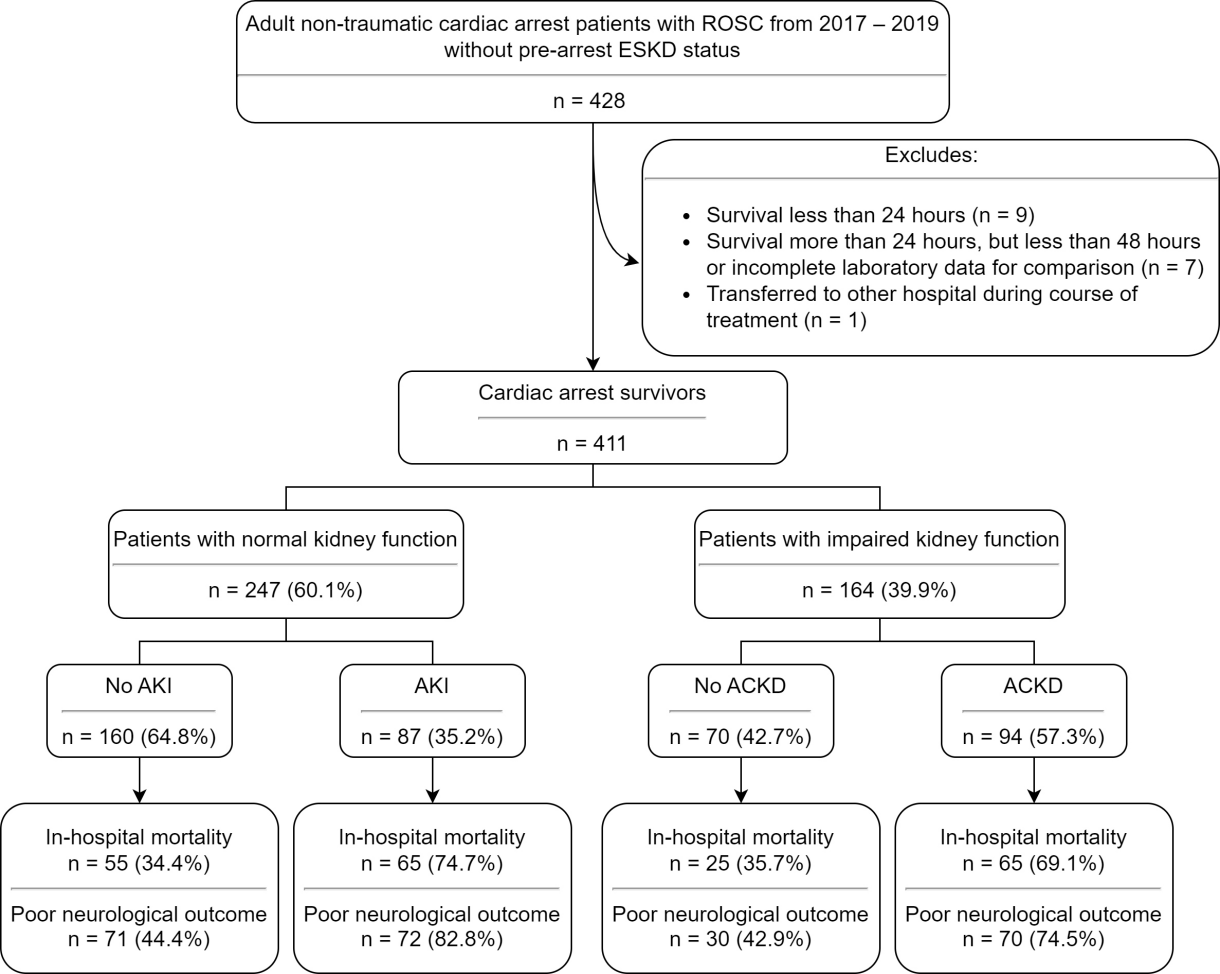
**Flowchart of patient enrollment**. Poor neurological outcome is defined as a Cerebral Performance Category score of 3 to 5. ACKD, acute on chronic kidney disease; AKI, acute kidney injury; ESKD, end-stage kidney disease; ROSC, return-of-spontaneous-circulation.

This study included all adult patients with nontraumatic cardiac arrest at the NTUH ED who underwent successful resuscitation and did not have pre-arrest end-stage kidney disease (ESKD) from January 2017 to December 2019. After excluding individuals who did not survive beyond 48 h (n = 9), those with incomplete data (n = 7), and those transferred to another hospital during treatment (n = 1), a total of 411 patients were included for analysis. These patients were further grouped according to their baseline serum creatinine levels at ROSC [16] into creatinine ≤1.5 mg/dL as the normal kidney function (NKF) group (n = 247) and creatinine >1.5 mg/dL as the impaired kidney function (IKF) group (n = 164) (Fig. 1).

### 3.2 Patient Characteristics and AKI

Within the cohort of 411 patients, the median age was 67 years, with a male predominant majority (71.3%). Among them, 181 (44.0%) patients developed acute kidney injury within 48 h after ROSC, with more than half of these cases eventually requiring KRT (n = 108, 59.7%), resulting in a mortality rate of 71.8%. A comparison of the characteristics, cardiac arrest events, postcardiac arrest interventions, and examinations between patients with and without AKI is presented in Table 1. Among patients with AKI, a higher prevalence of preexisting kidney disease (8.7% vs. 16.6%, *p* = 0.022), anemia (35.4% vs. 54.7%, *p* < 0.001), and malignancy (14.8% vs. 23.2%, *p* = 0.030) was observed. For cardiac arrest events, instances of repeated CPR (13.5% vs. 24.3%, *p* = 0.007) were more frequent among patients with AKI, whereas patients without AKI were more likely to exhibit an initial shockable rhythm (43.0% vs. 30.4%, *p* = 0.010) and receive less than 3 mg of epinephrine (83.9% vs. 66.9%, *p* < 0.001). Patients who underwent intra-aortic balloon pump (*p* = 0.020) and ECMO placement (*p* < 0.001) showed a stronger association with AKI, while patients with higher Glasgow Coma Scale scores at ROSC (*p* = 0.022) and subsequently more likely to receive TTM (*p* = 0.024) were less likely to develop AKI. Patients with AKI had lower hemoglobin concentrations (*p* < 0.001), pH levels (*p* = 0.006), bicarbonate levels (*p* = 0.015), and $O_{\text{2}}$/${\mathrm{FiO}}_{\text{2}}$ ratio (*p* < 0.001), along with higher levels of creatinine (*p* < 0.001), sodium (*p* = 0.011), potassium (*p* = 0.016), Troponin-T (*p* < 0.001), and LA (*p* < 0.001).

**Table 1.** **Characteristics between patients with and without AKI**.

		Overall	No AKI	AKI	*p*-value
		n = 411	n = 230	n = 181	
Male		293 (71.3)	168 (73.0)	125 (69.1)	0.382
Age >65 years		220 (53.5)	115 (50.0)	105 (58.0)	0.112
Age, years		67 (56–78)	66 (56–77)	68 (57–80)	0.094
Underlying characteristics					
Hypertension		199 (48.4)	109 (47.4)	90 (49.7)	0.691
DM		118 (28.7)	60 (26.1)	58 (32.0)	0.190
CAD		83 (20.2)	42 (18.3)	41 (22.7)	0.322
Heart failure		51 (12.4)	24 (10.4)	27 (14.9)	0.178
VHD		17 (4.1)	8 (3.5)	9 (5.0)	0.466
Arrhythmia		66 (16.1)	30 (13.0)	36 (19.9)	0.078
Kidney disease		50 (12.2)	20 (8.7)	30 (16.6)	0.022
Anemia		179 (43.9)	81 (35.4)	98 (54.7)	<0.001
CVA		40 (9.7)	24 (10.4)	16 (8.8)	0.619
Dementia		18 (4.4)	8 (3.5)	10 (5.5)	0.340
Bedridden		21 (5.1)	10 (4.3)	11 (6.1)	0.501
Malignancy		76 (18.5)	34 (14.8)	42 (23.2)	0.030
Cardiac Arrest Events					
Cardiac arrest location					
OHCA		294 (71.5)	173 (75.2)	121 (66.9)	0.062
Witnessed collapse		359 (87.3)	202 (87.8)	157 (86.7)	0.767
Initial shockable rhythm		154 (37.5)	99 (43.0)	55 (30.4)	0.010
Total CPR duration		17 (6–30)	18 (7–33)	17 (6–28)	0.248
	CPR >10 min	270 (65.7)	153 (66.5)	117 (64.6)	0.690
Epinephrine <3 mg		314 (76.4)	193 (83.9)	121 (66.9)	<0.001
Repeated CPR		75 (18.2)	31 (13.5)	44 (24.3)	0.007
Cardiogenic arrest		228 (55.5)	131 (57.0)	97 (53.6)	0.549
	ACS	107 (46.7)	63 (48.1)	44 (44.9)	0.005
	Arrhythmia	87 (38.0)	57 (43.5)	30 (30.6)	—
	Heart failure	9 (3.9)	4 (3.1)	5 (5.1)	—
	Others	26 (11.4)	7 (5.3)	19 (19.4)	—
Post-cardiac arrest events within 24 h after ROSC					
GCS M ≥2		237 (57.7)	144 (62.6)	93 (51.4)	0.022
Lowest MAP		73 (64–84)	76 (69–89)	69 (60–79)	<0.001
	MAP ≥65 mmHg	296 (72.0)	187 (81.3)	109 (60.2)	<0.001
${\mathrm{VIS}}_{\text{max}}$		9.5 (0–38.3)	3.8 (0–21.1)	21.9 (0–67.5)	<0.001
	No ${\mathrm{VIS}}_{\text{max}}$	155 (37.9)	102 (44.5)	53 (29.4)	<0.001
	Low ${\mathrm{VIS}}_{\text{max}}$ ≤30	139 (34.0)	90 (39.3)	49 (27.2)	—
	High ${\mathrm{VIS}}_{\text{max}}$ >30	117 (28.1)	38 (16.5)	79 (43.6)	—
TTM		150 (36.5)	95 (41.3)	55 (30.4)	0.024
IABP		48 (11.7)	19 (8.3)	29 (16.0)	0.020
ECMO		92 (22.4)	31 (13.5)	61 (33.7)	<0.001
Emergent CAG		145 (35.3)	85 (37.0)	60 (33.1)	0.467
Contrasted computed tomography scan		284 (69.1)	157 (68.3)	127 (70.2)	0.747
Laboratory Results at ROSC					
Hemoglobin, g/dL		13.2 (10.8–15.1)	13.6 (11.7–15.2)	12.1 (9.8–14.9)	<0.001
Creatinine, mg/dL		1.4 (1.0–1.8)	1.3 (1.0–1.6)	1.5 (1.1–2.2)	<0.001
Troponin-T, ng/L		34.8 (118.6–1488.0)	273.5 (96.4–956.3)	711.7 (139.7–3733.5)	<0.001
Lactic acid		6.0 (3.3–10.0)	4.2 (2.5–7.4)	8.8 (5.1–14.6)	<0.001
	LA <5 mmol/L	195 (47.4)	140 (60.9)	55 (30.4)	<0.001
	LA 5–10 mmol/L	113 (27.5)	58 (25.2)	55 (30.4)	—
	LA >10 mmol/L	103 (25.1)	32 (13.9)	71 (39.2)	—
pH value		7.35 (7.26–7.41)	7.36 (7.27–7.43)	7.33 (7.24–7.40)	0.006
${\mathrm{HCO}}_{3}$, mmol/L		18.7 (15.6–22.2)	19.3 (16.1–22.9)	17.9 (15.3–21.4)	0.015
$O_{\text{2}}$/${\mathrm{FiO}}_{\text{2}}$ ratio		234.9 (110.2–418.1)	302.4 (157.4–465.8)	171.4 (88.6–339.7)	<0.001
Outcomes					
KRT		108 (26.3)	0	108 (59.7)	<0.001
Mortality		210 (51.1)	80 (34.8)	130 (71.8)	<0.001
Poor neurological ${\mathrm{outcome}}^{1}$		243 (59.1)	101 (43.9)	142 (78.5)	<0.001

Data presented as no. (%) or as median (interquartile range (IQR)). ^1^Cerebral Performance Category score of 3 to 5 was considered a poor neurological outcome. ACS, acute coronary syndrome; CAD, coronary artery disease; CAG, coronary angiogram; CPR, cardiopulmonary resuscitation; CVA, cerebrovascular accident; DM, diabetes mellitus; ECMO, extracorporeal membrane oxygenation; GCS M, Glasgow Coma Scale motor component; IABP, intra-aortic balloon pump; MAP, mean arterial pressure; OHCA, out-of-hospital cardiac arrest; ROSC, return of spontaneous circulation; KRT, kidney replacement therapy; TTM, targeted temperature management; VHD, valvular heart disease; ${\mathrm{VIS}}_{\text{max}}$, maximum vasoactive-inotropic score; AKI, acute kidney injury; LA, lactic acid; ${\mathrm{FiO}}_{\text{2}}$, fraction of inspired oxygen.

**Fig. 2.**
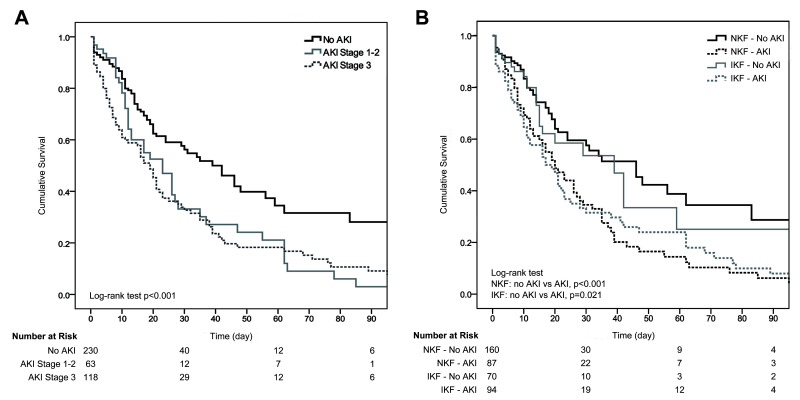
**Survival curves between patients with and without AKI**. (A) Comparison for overall patients. (B) Comparison according to baseline kidney function. AKI, acute kidney injury; NKF, normal kidney function; IKF, impaired kidney function.

In terms of hemodynamic status, patients with AKI had a significantly lower MAP (*p* < 0.001) and higher ${\mathrm{VIS}}_{\text{max}}$ (*p* < 0.001), with the majority of these patients receiving a high ${\mathrm{VIS}}_{\text{max}}$ >30. Approximately two-thirds of these patients necessitated KRT during hospitalization. Compared with patients without AKI, those with AKI stages 1–2 had a two-fold higher in-hospital mortality rate and an increased occurrence of poor neurological outcomes. Furthermore, patients with AKI stages 1–2 and AKI stage 3 were shown to have a lower 90-day survival rate than patients without AKI (Fig. 2A).

### 3.3 AKI Stratified by Baseline Kidney Function

There were 247 patients with NKF and 164 with IKF, of which 87 (35.2%) and 94 (57.3%) developed AKI, respectively. A comparison of the patient characteristics, cardiac arrest events, post-cardiac arrest interventions, and examinations between patients with and without AKI according to NKF and IKF are presented in **Supplementary Tables 1,2**, respectively.

Among patients with NKF, those without AKI had a higher proportion of patients with OHCA, initial shockable rhythm, and epinephrine use less than 3 mg. Conversely, patients with AKI had a higher frequency of repeated CPR. Furthermore, compared with patients without AKI, those with AKI received higher ${\mathrm{VIS}}_{\text{max}}$, had an increased incidence of ECMO placement, and exhibited elevated Troponin-T and LA levels, as well as lower MAP, hemoglobin concentration, pH value, and $O_{\text{2}}$/${\mathrm{FiO}}_{\text{2}}$ ratio. Among patients with IKF, the proportion of anemia was higher in patients with AKI. Similar patterns were noted in patients with NKF concerning epinephrine doses, ECMO placement, and laboratory results, including a lower MAP and higher ${\mathrm{VIS}}_{\text{max}}$ in patients with AKI. Regardless of baseline kidney function, patients with AKI in both the NKF and IKF groups showed higher mortality and poorer 90-day survival outcomes than those without AKI (Fig. 2B).

### 3.4 AKI and Outcomes

Compared with patients without AKI, those who developed AKI had significantly higher mortality rates (34.8% vs. 71.8%, adjusted OR [aOR] 5.40, 95% CI 3.36–8.69, *p* < 0.001) and poor neurological outcomes (43.9% vs. 78.5%, aOR 5.70, 95% CI 3.45–9.43, *p* < 0.001) upon discharge. Moreover, when stratified by AKI severity, patints with both AKI stages 1–2 and AKI stage 3 demonstrated higher mortality rates (aOR 3.85, 95% CI 2.00–7.42, *p* < 0.001 and aOR 6.54, 95% CI 3.76–11.38, *p* < 0.001, respectively) and poor neurological outcomes (aOR 3.90, 95% CI 1.96–7.76, *p* < 0.001 and aOR 7.17, 95% CI 3.93–13.05, *p* < 0.001, respectively), indicating a rising risk of adverse outcomes with increasing severity of kidney injury. These findings were consistently observed in both the NKF and IKF groups (Table 2).

**Table 2.** **Comparison of outcomes by AKI severity**.

Outcomes	No AKI	AKI	${\mathrm{aOR}}^{1}$ (95% CI)	AKI Stage 1–2	${\mathrm{aOR}}^{1}$ (95% CI)	AKI Stage 3	${\mathrm{aOR}}^{1}$ (95% CI)
Overall							
Mortality	80/230 (34.8%)	130/181 (71.8%)	5.40 (3.36–8.69)**	41/63 (65.1%)	3.85 (2.00–7.42)**	89/118 (75.4%)	6.54 (3.76–11.38)**
Poor neurological outcome	101/230 (43.9%)	142/181 (78.5%)	5.70 (3.45–9.43)**	45/63 (71.4%)	3.90 (1.96–7.76)**	97/118 (82.2%)	7.17 (3.93–13.05)**
Normal Kidney Function							
Mortality	55/160 (34.4%)	65/87 (74.7%)	5.86 (3.07–11.22)**	28/42 (66.7%)	3.80 (1.70–8.49)*	37/45 (82.2%)	9.42 (3.83–23.22)**
Poor neurological outcome	71/160 (44.4%)	72/87 (82.8%)	6.92 (3.36–14.25)**	31/42 (73.8%)	3.94 (1.66–9.37)*	41/45 (91.1%)	14.68 (4.67–46.13)**
Impaired Kidney Function							
Mortality	25/70 (35.7%)	65/94 (69.1%)	4.24 (2.34–10.50)**	13/21 (61.9%)	3.84 (1.22–12.12)*	52/73 (71.2%)	5.34 (2.41–11.81)**
Poor neurological outcome	30/70 (42.9%)	70/94 (74.5%)	5.26 (2.40–11.48)**	14/21 (66.7%)	3.75 (1.15–12.24)*	56/73 (76.7%)	5.83 (2.53–13.46)**

No AKI as a reference group. Cerebral Performance Category score 3 to 5 was considered a poor neurological outcome. ^1^ adjusted by age, witnessed collapse, initial shockable rhythm, TTM, total CPR duration. *p < 0.05, **p < 0.001. AKI, acute kidney injury; aOR, adjusted odds ratio; CI, confidence interval; OR, odds ratio; TTM, targeted temperature management; CPR, cardiopulmonary resuscitation.

### 3.5 ${\mathrm{VIS}}_{\text{max}}$ and AKI Development

Patients with a high ${\mathrm{VIS}}_{\text{max}}$ exhibited a nearly three-fold increased risk of developing AKI compared to those who did not receive any vasopressor support (aOR 2.96, 95% CI 1.61–5.45, *p* < 0.001) (Table 3). When stratified by illness severity, a low ${\mathrm{VIS}}_{\text{max}}$ was associated with the development of AKI stages 1–2 (aOR 2.51, 95% CI 1.20–5.24, *p* < 0.05), whereas a high ${\mathrm{VIS}}_{\text{max}}$ was linked to the occurrence of AKI stage 3 (aOR 2.46, 95% CI 1.28–4.75, *p* < 0.05). Additionally, a low ${\mathrm{VIS}}_{\text{max}}$ was correlated with a reduced likelihood of requiring KRT (aOR 0.39, 95% CI 0.20–0.78, *p* < 0.05), while a higher ${\mathrm{VIS}}_{\text{max}}$ was associated with higher rates of KRT (aOR 1.86, 95% CI 0.99–3.50, *p* = 0.056).

**Table 3.** **The impact of ${\mathrm{VIS}}_{\text{𝐦𝐚𝐱}}$ on AKI development**.

Groups	${\mathrm{VIS}}_{\text{max}}$	AKI	AKI Stage 1–2	AKI Stage 3
		aOR (95% CI)	aOR (95% CI)	aOR (95% CI)
${\mathrm{Overall}}^{1}$				
	No ${\mathrm{VIS}}_{\text{max}}$	—	—	—
	Low ${\mathrm{VIS}}_{\text{max}}$ ≤30	0.92 (0.53–1.62)	2.51 (1.20–5.24)*	0.46 (0.23–0.92)*
	High ${\mathrm{VIS}}_{\text{max}}$ >30	2.96 (1.61–5.45)**	1.75 (0.78–3.91)	2.46 (1.28–4.75)*
${\mathrm{NKF}}^{2}$				
	No ${\mathrm{VIS}}_{\text{max}}$	—	—	—
	Low ${\mathrm{VIS}}_{\text{max}}$ ≤30	1.10 (0.50–2.40)	4.81 (1.74–13.29)*	0.20 (0.06–0.63)*
	High ${\mathrm{VIS}}_{\text{max}}$ >30	2.67 (1.21–5.88)*	3.20 (1.11–9.20)*	1.33 (0.52–3.36)
${\mathrm{IKF}}^{3}$				
	No ${\mathrm{VIS}}_{\text{max}}$	—	—	—
	Low ${\mathrm{VIS}}_{\text{max}}$ ≤30	0.81 (0.36–1.81)	0.83 (0.27–2.59)	0.89 (0.38–2.12)
	High ${\mathrm{VIS}}_{\text{max}}$ >30	5.23 (1.92–14.25)*	0.76 (0.22–2.62)	5.18 (2.01–13.35)*

No ${\mathrm{VIS}}_{\text{max}}$ as a reference group. *p < 0.05, **p < 0.001. ^1^adjusted for sex, age, anemia, malignancy, OHCA, initial shockable rhythm, repeated CPR, total CPR duration, ECMO, TTM, elevated baseline creatinine, and LA. ^2^adjusted for sex, age, OHCA, initial shockable rhythm, repeated CPR, total CPR duration, ECMO, and LA. ^3^adjusted for sex, age, anemia, OHCA, total CPR duration, and ECMO. AKI, acute kidney injury; CI, confidence interval; CPR, cardiopulmonary resuscitation; ECMO, extracorporeal membrane oxygenation; IKF, impaired kidney function; LA, lactic acid; NKF, normal kidney function; OHCA, out-of-hospital cardiac arrest; aOR, adjusted odds ratio; TTM, targeted temperature management; ${\mathrm{VIS}}_{\text{max}}$, maximum vasoactive inotropic score.

Regardless of baseline kidney function, a higher ${\mathrm{VIS}}_{\text{max}}$ was associated with a higher AKI risk (aOR 2.67, 95% CI 1.21–5.88, *p* < 0.05 and aOR 5.23, 95% CI 1.92–14.25, *p* < 0.05, respectively). Among patients with NKF, both low and high ${\mathrm{VIS}}_{\text{max}}$ increased the risk of AKI stage 1–2 (aOR 4.81, 95% CI 1.74–13.29, *p* < 0.05 and aOR 3.20, 95% CI 1.11–9.20, *p* < 0.05, respectively). On the other hand, among patients with IKF, a high ${\mathrm{VIS}}_{\text{max}}$ increased the risk of AKI stage 3 (aOR 5.18, 95% CI 2.01–13.35, *p* < 0.05).

## 4. Discussion

In this study, patients who developed AKI during hospitalization exhibited higher mortality rates and increased incidence of poor neurological outcomes, and the risk increases as the severity of illness escalates. Furthermore, patients with vasopressor dependency during the early postarrest period were more prone to developing early AKI following ROSC, irrespective of their baseline kidney function at the time of ROSC. Vasopressor use in patients with NKF was correlated with the development of AKI stages 1-2, whereas increased vasopressor use heightened the likelihood of developing severe AKI in patients with baseline IKF.

Patients who have undergone resuscitation are at risk of developing AKI [1, 3, 6], as evidenced by the high incidence in our cohort. Approximately two-thirds of the patients eventually progressed to severe AKI and required dialysis; these patients also experienced double the rate of in-hospital mortality and poor neurological outcomes than patients without AKI. These patterns were consistent across both NKF and IKF groups, showcasing significantly higher mortality rates and poor neurological outcomes among patients with AKI, with the risk increasing as the severity of kidney injury increases, in alignment with previous literature [4, 25, 26]. Notably, Acosta-Ochoa *et al*. [14] divided all hospitalized patients with AKI into previously NKF or IKF groups and then classified them according to the KDIGO-2012 criteria; they observed that AKI severity was associated with in-hospital mortality, independent of baseline kidney function. In comparison to the IKF group, patients with NFK demonstrated higher in-hospital mortality in accordance with AKI severity, suggesting a link between in-hospital mortality and the extent of AKI rather than baseline kidney function [14, 17]. Even minor fluctuations in the serum creatinine level were strongly associated with adverse outcomes [17, 27]. Therefore, the development and severity of AKI play a pivotal role in determining the prognosis of cardiac arrest survivors.

Cardiovascular compromise during and after cardiac arrest, particularly kidney hypoperfusion, significantly contributes to AKI development [1, 3, 13]. Vasopressor use following ROSC is a risk factor for AKI and increases the risk of long-term KRT [3]; moreover, the severity of AKI has been associated with the increased number of postarrest vasoactive agents used [9]. However, previous studies did not quantify vasopressor dependency, despite evidenced to be a good predictor of in-hospital mortality and AKI development [12, 13]. The degree of cardiovascular support, quantified using the VIS, reflects the severity of the hemodynamic disturbance and has been considered an accurate predictor for short-term mortality and morbidity in patients undergoing cardiovascular surgery [10, 13, 28]. The VIS score has a higher predictive accuracy for short-term morbidity than the Acute Physiology and Chronic Health Evaluation II and exhibited similar performance with the Sequential Organ Failure Score in patients undergoing cardiac surgery [10, 28]. The 24 h-peak VIS is also a suitable scoring system for predicting in-hospital mortality in patients with OHCA, with an optimal cutoff value of 33.3 [12]. Our study aimed to assess the relationships between vasopressor dependence denoted as ${\mathrm{VIS}}_{\text{max}}$, and adverse outcomes. It was shown that patients who had received a higher dose of vasopressors had an increased AKI risk. Higher doses of catecholamines have been shown to induce organ damage and immune-mediated injuries, thereby potentially increasing the incidence of AKI [13, 29, 30]. This can not only help predict prognosis but also serve as a tool to help guide treatment [13].

Among surgical or septic patients, those with IKF are at greater risk of AKI and adverse outcomes than those with NKF [31, 32]. In our study, AKI severity increased as ${\mathrm{VIS}}_{\text{max}}$ increased in patients with baseline NKF or IKF. Among patients with NKF, vasopressor administration, irrespective of dose, was associated with an increased risk of AKI stages 1–2, particularly with low ${\mathrm{VIS}}_{\text{max}}$. Low ${\mathrm{VIS}}_{\text{max}}$ was inversely associated with severe AKI development, while high ${\mathrm{VIS}}_{\text{max}}$ did not show a significant association with severe AKI. Among patients with IKF, low ${\mathrm{VIS}}_{\text{max}}$ did not increase AKI risk, irrespective of AKI severity. Only high ${\mathrm{VIS}}_{\text{max}}$ was associated with the development of severe AKI. These findings suggest that high vasopressor dependency is associated with AKI stages 1–2 in patients with NKF and AKI stage 3 in patients with IKF. It is proposed that a pre-existing compromised kidney is more susceptible to acute injury due to chronic comorbidities, impaired autoregulation, and increased exposure to nephrotoxic injuries [17, 33]. Similar observations were made in septic patients, where vasopressin use improved kidney function and reduced the need for KRT in patients at risk of AKI but not those who had already sustained significant kidney injury [34]. Recognizing patients at risk for AKI development based on their baseline kidney function and degree of vasopressor dependence can allow for more stringent monitoring of signs and symptoms of AKI and the selection of appropriate management during the early postarrest period.

### Limitations

This study had several limitations. Firstly, due to its retrospective nature, selection bias was unavoidable, and unidentified confounding factors may be present. As a result of the study design, there is a lack of a strict protocol for the use of vasoactive and inotropic medications, which may be dependent on the attending physicians’ decisions at the time. Additionally, certain data may not be available for all patients; hence, not all possible factors that may contribute to AKI development were taken into account for analysis. Secondly, the definition of AKI in this study was based on serum creatinine alone, and not including urine output may not reflect the true classification of this population. Thirdly, our findings may have limited generalizability depending on the different hospital protocols in terms of postarrest care strategies, which may influence AKI development and overall outcomes. Lastly, only short-term mortality and neurological recovery at hospital discharge were assessed. It would be prudent for future well-designed studies to explore long-term outcomes after discharge.

## 5. Conclusions

${\mathrm{VIS}}_{\text{max}}$ during the early postarrest period is an independent predictor of the development and severity of early AKI following ROSC, regardless of baseline kidney function at ROSC. Our findings should be validated in large-scale prospective studies.

## Data Availability

The datasets generated and/or analysed during the current study are available from the corresponding author on reasonable request.

## Abbreviations

AKI, acute kidney injury; CA, cardiac arrest; CPC, Cerebral Performance Category; CPR, cardiopulmonary resuscitation; ECMO, extracorporeal membrane oxygenation; IKF, impaired kidney function; KDIGO, Kidney Disease: Improving Global Outcomes; LA, lactic acid; MAP, mean arterial pressure; NKF, normal kidney function; OHCA, out-of-hospital cardiac arrest; ROSC, return of spontaneous circulation; KRT, kidney replacement therapy; TTM, targeted temperature management; VIS, vasoactive-inotropic score; ${\mathrm{VIS}}_{\text{max}}$, maximum VIS in 24 hours.

## Supplementary Material

Supplementary material associated with this article can be found, in the online version, at https://doi.org/10.31083/j.rcm2501004.
