# Supplementary material for: Impact of Postarrest Vasoactive-Inotropic Score on Acute Kidney Injury in Cardiac Arrest Survivors: A Retrospective Cohort Study
Source: Rev Cardiovasc Med. 2024 Jan 4;25(1):4. doi: 10.31083/j.rcm2501004 (PMC11262340; doi:10.31083/j.rcm2501004)
Supplement: Supplementary file 1 [file 2153-8174-25-1-004-s1.docx]

**Supplementary Table 1. Patient characteristics between patients with and without AKI with previously normal kidney function**

|  | **Overall** | | | **No AKI** | **AKI** | ***p-value*** | | |  |
| --- | --- | --- | --- | --- | --- | --- | --- | --- | --- |
|  | **n = 247** | | | **n = 160** | **n = 87** |  | | |  |
| Male | 160 (64.8) | | | 110 (68.8) | 50 (57.5) | 0.094 | | |  |
| Age > 65 years | 121 (49.0) | | | 74 (46.2) | 47 (54.0) | 0.287 | | |  |
| Age, years | 65 (54-76) | | | 64 (55-74) | 66 (53-80) | 0.242 | | |  |
| **Underlying Characteristics** | | | | | | | | | |
| Hypertension | 116 (47.0) | | | 76 (47.5) | 40 (46.0) | 0.894 | | |  |
| DM | 60 (24.3) | | | 35 (21.9) | 25 (28.7) | 0.277 | | |  |
| CAD | 39 (15.8) | | | 25 (15.6) | 14 (16.1) | 1.000 | | |  |
| Heart failure | 15 (6.1) | | | 9 (5.6) | 6 (6.9) | 0.782 | | |  |
| VHD | 8 (3.2) | | | 4 (2.5) | 4 (4.6) | 0.457 | | |  |
| Arrhythmia | 29 (11.7) | | | 15 (9.4) | 14 (16.1) | 0.147 | | |  |
| Kidney disease | 8 (3.2) | | | 5 (3.1) | 3 (3.4) | 1.000 | | |  |
| Anemia | 51 (20.6) | | | 32 (20.0) | 19 (21.8) | 0.744 | | |  |
| CVA | 25 (10.1) | | | 17 (10.6) | 8 (9.2) | 0.827 | | |  |
| Dementia | 9 (3.6) | | | 6 (3.8) | 3 (3.4) | 1.000 | | |  |
| Bedridden | 8 (3.2) | | | 5 (3.1) | 3 (3.4) | 1.000 | | |  |
| Malignancy | 13 (5.3) | | | 7 (4.4) | 6 (6.9) | 0.389 | | |  |
| **Cardiac Arrest Events** | | | | | | | | | |
| Cardiac arrest location | | |  |  |  |  |  |  |  |
| OHCA | | 175 (70.9) | | 121 (75.6) | 54 (62.1) | 0.025 | | |  |
| Witnessed collapse | | 214 (86.6) | | 139 (86.9) | 75 (86.2) | 1.000 | | |  |
| Initial shockable rhythm | | 93 (37.7) | | 70 (43.8) | 23 (26.4) | 0.009 | | |  |
| Total CPR duration | | 15 (5-30) | | 16 (5-34) | 13 (5-26) | 0.206 | | |  |
| CPR >10 min | | 154 (62.3) | | 103 (64.4) | 51 (58.6) | 0.373 | | |  |
| Epinephrine <3 mg | | 194 (78.5) | | 136 (85.0) | 58 (66.7) | 0.001 | | |  |
| Repeated CPR | | 46 (18.6) | | 19 (11.9) | 27 (31.0) | 0.001 | | |  |
| Cardiogenic arrest | | 132 (53.4) | | 87 (54.4) | 45 (51.7) | 0.691 | | |  |
| **Post-Cardiac Arrest Events within 24-h after ROSC** | | | | | | | |  |  |
| GCS M ≥2 | | 150 (60.7) | | 104 (65.0) | 46 (52.9) | 0.062 | | |  |
| Lowest MAP | | 76 (66-85) | | 77 (70-91) | 70 (59-82) | <0.001 | | |  |
| MAP ≥65 mmHg | | 190 (76.9) | | 136 (85.0) | 54 (62.1) | <0.001 | | |  |
| VIS_max_ | | 9.4 (0-37.3) | | 3.3 (0-22.4) | 27.7 (2.1-83.7) | <0.001 | | |  |
| No VIS_max_ | | 95 (38.8) | | 74 (46.5) | 21 (24.4) | <0.001 | | |  |
| Low VIS_max_ ≤30 | | 81 (33.1) | | 56 (35.2) | 25 (29.1) | — | | |  |
| High VIS_max_ >30 | | 69 (28.2) | | 29 (18.2) | 40 (46.5) | — | | |  |
| TTM | | 100 (40.5) | | 69 (43.1) | 31 (35.6) | 0.279 | | |  |
| IABP | | 22 (8.9) | | 12 (7.5) | 10 (11.5) | 0.351 | | |  |
| ECMO | | 47 (19.0) | | 19 (11.9) | 28 (32.2) | <0.001 | | |  |
| Emergent CAG | | 83 (33.6) | | 56 (35.0) | 27 (31.0) | 0.574 | | |  |
| Contrasted CT scan | | 181 (73.3) | | 116 (72.5) | 65 (74.7) | 0.765 | | |  |
| **Laboratory Results at ROSC** | | | | | | | |  |  |
| Hemoglobin, g/dL | | 13.2 (11.4-15.1) | | 13.6 (11.8-15.1) | 12.4 (10.2-15.1) | 0.014 | | |  |
| Creatinine, mg/dL | | 1.1 (0.9-1.3) | | 1.1 (0.9-1.3) | 1.1 (0.8-1.3) | 0.596 | | |  |
| Troponin-T, ng/L | | 332.7 (111.8-1277.0) | | 273.5 (96.4-951.1) | 735.8 (139.9-2197) | 0.008 | | |  |
| Lactic acid | | 4.8 (3.1-8.6) | | 4.1 (2.5-6.5) | 8.5 (5.4-12.8) | <0.001 | | |  |
| LA <5 mmol/L | | 129 (52.2) | | 102 (63.7) | 27 (31.0) | <0.001 | | |  |
| LA 5-10 mmol/L | | 67 (27.1) | | 40 (25.0) | 27 (31.0) | — | | |  |
| LA >10 mmol/L | | 51 (20.6) | | 18 (11.2) | 33 (37.9) | — | | |  |
| pH value | | 7.35 (7.26-7.41) | | 7.36 (7.30-7.43) | 7.29 (7.23-7.36) | 0.001 | | |  |
| HCO3, mmol/L | | 18.9 (15.9-22.6) | | 19.1 (16.4-22.9) | 18.1 (15.4-22.2) | 0.191 | | |  |
| O2/FiO2 ratio | | 264.6 (134.2-421.0) | | 304.8 (175.6-469.2) | 229.0 (94.7-329.0) | 0.003 | | |  |
| **Outcomes** | | |  |  |  |  |  |  |  |
| KRT | | | 42 (17.0) | 0 | 42 (48.3) | <0.001 |  |  |  |
| Mortality | | | 120 (48.6) | 55 (34.4) | 65 (74.7) | <0.001 |  |  |  |
| Poor neurological outcome^1^ | | | 143 (57.9) | 71 (44.4) | 72 (82.8) | <0.001 |  |  |  |

Data presented as no. (%) or as median (IQR).

^1^Cerebral Performance Category score of 3 to 5 was considered as poor neurological outcome.

APACHE-II: acute physiology and chronic health evaluation; CAD, coronary artery disease; CAG, coronary angiogram; CPR, cardiopulmonary resuscitation; CVA, cerebrovascular accident; DM, diabetes mellitus; ECMO, extracorporeal membrane oxygenation; GCS M, Glasgow Coma Scale motor component; IABP, intra-aortic balloon pump; IV, intravenous; MAP, mean arterial pressure; OHCA, out-of-hospital cardiac arrest; ROSC, return of spontaneous circulation; KRT, kidney replacement therapy; TTM, targeted temperature management; VHD, valvular heart disease; VIS_max_, maximum vasoactive-inotropic score.

**Supplementary Table 2. Patient characteristics between patients with and without AKI with impaired kidney function**

|  | **Overall** | | | | **No AKI** | | **AKI** | | | | ***p*-*value*** | | |  |  |
| --- | --- | --- | --- | --- | --- | --- | --- | --- | --- | --- | --- | --- | --- | --- | --- |
|  | **n = 164** | | | | **n = 70** | | **n = 94** | | | |  | | |  |  |
| Male | 133 (81.1) | | | | 58 (82.9) | | 75 (79.8) | | | | 0.690 | | |  |  |
| Age > 65 years | 99 (60.4) | | | | 41 (58.6) | | 58 (61.7) | | | | 0.748 | | |  |  |
| Age, years | 69 (57-80) | | | | 69 (56-79) | | 70 (58-80) | | | | 0.804 | | |  |  |
| **Underlying Characteristics** | | | | | | | | | | | | | |  |  |
| Hypertension | 83 (50.6) | | | | 33 (47.1) | | 50 (53.2) | | | | 0.528 | | |  |  |
| DM | 58 (35.4) | | | | 25 (35.7) | | 33 (35.1) | | | | 1.000 | | |  |  |
| CAD | 44 (26.8) | | | | 17 (24.3) | | 27 (28.7) | | | | 0.595 | | |  |  |
| Heart failure | 36 (22.0) | | | | 15 (21.4) | | 21 (22.3) | | | | 1.000 | | |  |  |
| VHD | 9 (5.5) | | | | 4 (5.7) | | 5 (5.3) | | | | 1.000 | | |  |  |
| Arrhythmia | 37 (22.6) | | | | 15 (21.4) | | 22 (23.4) | | | | 0.851 | | |  |  |
| Kidney disease | 42 (25.6) | | | | 15 (21.4) | | 27 (28.7) | | | | 0.366 | | |  |  |
| Anemia | 82 (50.3) | | | | 27 (38.6) | | 55 (59.1) | | | | 0.011 | | |  |  |
| CVA | 15 (9.1) | | | | 7 (10.0) | | 8 (8.5) | | | | 0.789 | | |  |  |
| Dementia | 9 (5.5) | | | | 2 (2.9) | | 7 (7.4) | | | | 0.303 | | |  |  |
| Bedridden | 6 (3.7) | | | | 3 (4.3) | | 3 (3.2) | | | | 0.701 | | |  |  |
| Malignancy | 8 (4.9) | | | | 3 (4.3) | | 5 (5.3) | | | | 1.000 | | |  |  |
| **Cardiac Arrest Events** | | | | | | | | | | | | | |  |  |
| Cardiac arrest location | | |  | |  | |  | | | |  | | |  |  |
| OHCA | | 119 (72.6) | | | 52 (74.3) | | 67 (71.3) | | | | 0.669 | | |  |  |
| Witnessed collapse | | 145 (88.4) | | | 63 (90.0) | | 82 (87.2) | | | | 0.631 | | |  |  |
| Initial shockable rhythm | | 61 (37.4) | | | 29 (41.4) | | 32 (34.0) | | | | 0.414 | | |  |  |
| Total CPR duration | | 19 (9-31) | | | 20 (10-33) | | 19 (9-28) | | | | 0.508 | | |  |  |
| CPR >10 min | | 116 (70.7) | | | 50 (71.4) | | 66 (70.2) | | | | 0.866 | | |  |  |
| Epinephrine <3 mg | | 120 (73.2) | | | 57 (81.4) | | 63 (67.0) | | | | 0.050 | | |  |  |
| Repeated CPR | | 29 (17.7) | | | 12 (17.1) | | 17 (18.1) | | | | 1.000 | | |  |  |
| Cardiogenic arrest | | 96 (58.5) | | | 44 (62.9) | | 52 (55.3) | | | | 0.342 | | |  |  |
| **Post-Cardiac Arrest Events within 24-h after ROSC** | | | | | | | | | | | | |  |  |  |
| GCS M ≥2 | | 87 (53.0) | | | 40 (57.1) | | 47 (50.0) | | | | 0.365 | | |  |  |
| Lowest MAP | | 71 (63-82) | | | 75 (65-89) | | 68 (60-76) | | | | 0.001 | | |  |  |
| MAP ≥65 mmHg | | 106 (64.6) | | | 51 (72.9) | | 55 (58.5) | | | | 0.057 | | |  |  |
| VIS_max_ | | 9.8 (0-38.9) | | | 4.6 (0-19.2) | | 19.1 (0-63.5) | | | | 0.005 | | |  |  |
| No VIS_max_ | | 60 (36.6) | | | 34 (40.0) | | 32 (34.0) | | | | <0.001 | | |  |  |
| Low VIS_max_ ≤30 | | 58 (35.4) | | | 34 (48.6) | | 24 (25.5) | | | | — | | |  |  |
| High VIS_max_ >30 | | 46 (28.0) | | | 8 (11.4) | | 38 (40.4) | | | | — | | |  |  |
| TTM | | 50 (30.5) | | | 26 (37.1) | | 24 (25.5) | | | | 0.125 | | |  |  |
| IABP | | 26 (15.9) | | | 7 (10.0) | | 19 (20.2) | | | | 0.087 | | |  |  |
| ECMO | | 45 (27.4) | | | 12 (17.1) | | 33 (35.1) | | | | 0.013 | | |  |  |
| Emergent CAG | | 62 (37.8) | | | 29 (41.4) | | 33 (35.1) | | | | 0.421 | | |  |  |
| Contrasted CT scan | | 103 (62.8) | | | 41 (58.6) | | 62 (66.0) | | | | 0.414 | | |  |  |
| **Laboratory Results at ROSC** | | | | | | | | | | | | |  |  |  |
| Hemoglobin, g/dL | | 12.7 (10.1-15.1) | | | 13.7 (10.9-15.4) | | 11.4 (9.4-14.8) | | | | 0.017 | | |  |  |
| Creatinine, mg/dL | | 2.1 (1.7-2.7) | | | 1.9 (1.6-2.5) | | 2.2 (1.7-2.9) | | | | 0.058 | | |  |  |
| Troponin-T, ng/L | | 393.7 (120.3-2247.5) | | | 291.6 (97.6-1113) | | 641.7 (139.4-6333) | | | | 0.022 | | |  |  |
| Lactic acid | | 7.5 (3.7-12.8) | | | 4.7 (2.5-9.3) | | 9.5 (5.3-14.9) | | | | <0.001 | | |  |  |
| LA <5 mmol/L | | 66 (40.2) | | | 38 (54.3) | | 28 (29.8) | | | | 0.003 | | |  |  |
| LA 5-10 mmol/L | | 46 (28.0) | | | 18 (25.7) | | 28 (29.8) | | | | — | | |  |  |
| LA >10 mmol/L | | 52 (28.0) | | | 18 (25.7) | | 28 (29.8) | | | | — | | |  |  |
| pH value | | 7.34 (7.26-7.42) | | | 7.35 (7.25-7.43) | | 7.34 (7.26-7.40) | | | | 0.758 | | |  |  |
| HCO3, mmol/L | | 18.3 (15.4-21.9) | | | 19.9 (15.4-22.5) | | 17.6 (15.4-20.9) | | | | 0.078 | | |  |  |
| O2/FiO2 ratio | | 206.0 (93.1-399.2) | | | 288.9 (133.1-432.4) | | 156.2 (83.4-353.5) | | | | 0.011 | | |  |  |
| **Outcomes** | | |  | | |  | |  | | | |  | | |  |
| KRT | | | 66 (40.2) | 0 | | | | | 66 (70.2) | <0.001 | | | | | |
| Mortality | | | 90 (54.9) | 25 (35.7) | | | | | 65 (69.1) | <0.001 | | | | | |
| Poor neurological outcome^1^ | | | 100 (61.0) | 30 (42.9) | | | | | 70 (74.5) | <0.001 | | | | | |

Data presented as no. (%) or as median (IQR).

^1^Cerebral Performance Category score of 3 to 5 were considered as poor neurological outcome.

APACHE-II: acute physiology and chronic health evaluation; CAD, coronary artery disease; CAG, coronary angiogram; CPR, cardiopulmonary resuscitation; CVA, cerebrovascular accident; DM, diabetes mellitus; ECMO, extracorporeal membrane oxygenation; GCS M, Glasgow Coma Scale motor component; IABP, intra-aortic balloon pump; IV, intravenous; MAP, mean arterial pressure; OHCA, out-of-hospital cardiac arrest; ROSC, return of spontaneous circulation; KRT, kidney replacement therapy; TTM, targeted temperature management; VHD, valvular heart disease; VIS_max_, maximum vasoactive-inotropic score.
